# Crude Plant Extracts and Their Anti-Inflammatory Potential in Oral Inflammatory Cell Models: A Systematic Review of In Vitro Studies

**DOI:** 10.3390/ijms262311253

**Published:** 2025-11-21

**Authors:** Issam Rasheed, Reinhard Gruber

**Affiliations:** 1 Department of Oral Biology, University Clinic of Dentistry, Medical University of Vienna, Sensengasse 2a, 1090 Vienna, Austria; 2 Department of Periodontology, School of Dental Medicine, University of Bern, 3010 Bern, Switzerland; 3 Austrian Cluster for Tissue Regeneration, 1200 Vienna, Austria

**Keywords:** plant extracts, herbal medicine, oral inflammation, oral mucositis, periodontitis, cytokines, anti-inflammatory, in vitro

## Abstract

Plants are a rich source of bioactive compounds with broad pharmaceutical potential, particularly for their anti-inflammatory properties. Oral inflammation underlies many local and systemic diseases, yet conventional anti-inflammatory drugs have adverse effects. Crude plant extracts offer promising, safer alternatives. This systematic review synthesizes evidence on the anti-inflammatory activity of whole plant extracts in vitro oral models of inflammation. It also highlights methodological considerations for improved reproducibility. PubMed searches following PRISMA guidelines identified studies using oral or periodontal cells stimulated with relevant inflammatory triggers and treated with crude plant extracts. Extracts from *Camellia sinensis*, *Salvia officinalis*, *Paeonia x suffruticosa*, *Houttuynia cordata*, *Theobroma cacao*, and others consistently reduced pro-inflammatory cytokines such as IL-6, IL-8, IL-1β, and TNF-α, with no reported cytotoxicity at the effective concentrations. Variations in model design, extract characterization, and stimulation protocols were noted. Overall, this review demonstrates that crude plant extracts effectively modulate oral inflammatory responses in vitro. Findings suggest a possible non-cytotoxic anti-inflammatory activity that requires further investigation and underscore the need for methodological standardization to advance clinical translation.

## 1. Introduction

Inflammation is a tightly regulated physiological process fundamental to host defense against pathogens, tissue injury, and other harmful stimuli [1]. Beyond its defensive role, inflammation interacts dynamically with host metabolism, the commensal microbiome, and resident immune cells, shaping tissue homeostasis and systemic health [2,3,4]. In the oral cavity, inflammation is central to a wide range of local and systemic disorders. Conditions such as pulpitis, periodontitis, and stomatitis are primarily driven by inflammatory processes, which, if uncontrolled, can lead to tissue destruction and contribute to systemic complications via circulating inflammatory mediators [5,6]. Although dental interventions—including pulpectomy, exodontia, and scaling—effectively manage acute and chronic dental inflammation, non-dental inflammatory conditions, such as mucositis and periodontitis, remain challenging. Pharmacological agents are frequently employed, yet their clinical use is often limited by their systemic side effects, cytotoxicity, or patient intolerance. This underscores the need for safe, effective, and accessible alternatives for controlling oral inflammation.

Plant materials have been used medicinally for centuries across various ethnic groups and geographical locations in the world [7]. Their continued use in under-resourced and geographically isolated populations highlights both their accessibility and cultural relevance, as well as their role where socioeconomic barriers to access to medical care exist [8,9]. In recent decades, plant extracts have gained renewed interest due to their pharmacological potency, relative safety, and cost-effectiveness [10]. Bioactive compounds—including flavonoids, polyphenols, terpenoids, and alkaloids—have been shown to modulate inflammatory pathways by influencing cytokine production, chemokine expression, and transcription factor activity. Fractionation and chromatography have enabled the isolation of specific bioactive compounds with anti-inflammatory activity, which are then evaluated in vitro and in vivo. Despite promising preclinical findings, clinical translation remains limited, and few standardized formulations are currently approved for oral inflammatory conditions.

Topical anti-inflammatory agents remain a cornerstone of oral disease management, such as in the management of recurrent aphthous stomatitis (RAS) and radiation-induced mucositis (RIM). For instance, clinical evidence supported the use of benzydamine mouthwashes for the symptomatic relief of RIM [11]. Nonetheless, *Aloe vera* demonstrated comparable effects to benzydamine in a clinical setting [12]. A recent systematic review and meta-analysis indicated that herbal-derived compounds from turmeric and sumac may outperform conventional therapies in specific contexts, reinforcing the therapeutic potential of plant-based interventions [13]. Nevertheless, the search for safer, more effective, and accessible alternatives continues [14].

Crude plant extracts are not inherently safe by virtue of being natural. Their toxicological profile varies with species, extraction method, concentration, and bioassay, necessitating careful toxicological evaluation. Global health bodies emphasize the need for systematic and vigilant safety assessment of herbal medicines [15,16]. Evidence from systematic reviews and toxicological evaluations indicates that most plant extracts exhibit favorable safety margins when used with traditional dosages [17,18]. Safety reporting remains key when investigating the biological activities of plant extracts.

Mechanistically, oral inflammation involves complex networks of pro-inflammatory mediators, including cytokines (IL-1β, IL-6, TNF-α), chemokines (CCL2, CCL5, CXCL family members), prostaglandins (PGE_2_), and enzymes such as COX-2. These mediators orchestrate immune cell recruitment, activation, and tissue remodeling, and their dysregulation contributes to pathological outcomes [19,20]. Plant-derived compounds can modulate these pathways by suppressing cytokine and chemokine expression, inhibiting transcription factors such as NF-κB and AP-1, reducing oxidative stress, and downregulating inflammatory enzymes [21]. In vitro models using fibroblasts, epithelial cells, keratinocytes, osteocytes, and immune cell co-cultures have been instrumental in elucidating these mechanisms, but the evidence remains fragmented and often extrapolated from non-oral systems.

Furthermore, methodological heterogeneity—including variations in extraction methods, concentrations, cell models, inflammatory stimuli, and assay endpoints—limits reproducibility and comparability across studies. A systematic synthesis of the current literature on crude plant extracts in oral in vitro models is therefore essential. Such a review can consolidate evidence on anti-inflammatory efficacy, identify reproducible effects, clarify molecular mechanisms, and highlight methodological gaps, ultimately informing both pre-clinical research and translational applications.

This systematic review aims to provide a comprehensive evaluation of the anti-inflammatory potential of whole plant extracts in oral in vitro models. It aims to summarize and review evidence of crude plant extracts in cell models of oral inflammation, characterizing the ethnomedicinal in these models for further mechanistic evaluation. Thus, acknowledging the practical and traditional use of herbal materials as accessible interventions, while identifying methodological limitations, promoting standardization and reproducibility in future research.

## 2. Materials and Methods

### 2.1. Search Strategy

This systematic review was designed according to the Preferred Reporting Items for Systematic Review and Meta-analysis (PRISMA) recommendations. Between April 2025 and August 2025, an electronic search was conducted in the PubMed database without time or language restrictions, and without any filters. The search string was developed using a combination of medical subject heading database (MeSH) terms and keywords connected with Boolean operators, to ensure a comprehensive collection of studies. The search string was as follows: ((“Inflammation”[Mesh]) OR (“Inflammation Mediators”[Mesh]) OR (“Stomatitis”[Mesh]) OR (“Mucositis”[Mesh]) OR (“Gingivitis”[Mesh]) OR (“Periodontitis”[Mesh]) OR (“Mouth Diseases”[Mesh]) OR (“Interleukins”[Mesh]) OR (“Interleukin-1”[Mesh]) OR (“Interleukin-1beta”[Mesh]) OR (“Interleukin-6”[Mesh]) OR (“Tumor Necrosis Factor-alpha”[Mesh]) OR (“Cytokines”[Mesh])) AND ((“Plant Extracts”[Mesh]) OR (“Drugs, Chinese Herbal”[Mesh])) AND ((“In Vitro Techniques”[Mesh]) OR (“Cell Culture Techniques”[Mesh]) OR (“gingival fibroblasts”) OR (“periodontal fibroblasts”) OR (“oral epithelial cells”) OR (“oral keratinocytes”)).

### 2.2. Study Selection and Eligibility Criteria

Initially, eligible studies were identified for compatibility with the aim of this systematic review. Thus, the screening of titles and abstracts for in vitro studies investigating the effects of plant extracts on inflammation was conducted. Following this, full-text screening was carried out, applying the eligibility criteria. Eligibility criteria were specified using the PICO criteria as follows: population was oral inflammatory cell models or in vitro oral inflammatory models, intervention was crude plant extracts regardless of the part of the plant used to obtain the extract, control was positive (inflammation-triggered cells) and negative (cells without stimulation), and outcome was the anti-inflammatory effects.

Inclusion criteria were studies that were in vitro studies using oral cell models or co-culture models of oral cells and immune cells. Only studies with direct inflammatory assay and the use of relevant inflammatory stimuli were included. Studies that utilized crude plant extracts alone or alongside their fractionated compounds were included if a separate reporting of outcomes of the crude plant extract existed. In addition, the criteria applied were studies with clear reporting of methods and analysis.

**Figure 1:**
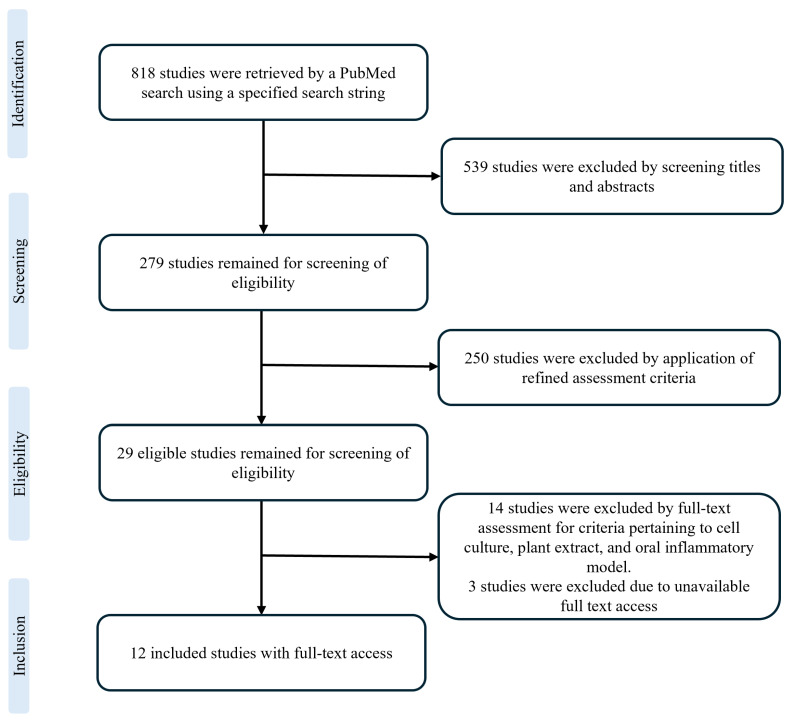
Flow diagram of study selection.

Exclusion based on study design was performed for review articles, in vivo studies, clinical human studies, and chemical characterization studies. Studies on isolated or fractionated compounds were excluded. Similarly, studies that employed non-oral cell types or immune cells alone were excluded. Additionally, studies that investigated the inflammatory and allergic effects of plant parts, such as pollen and dust, were excluded. Figure 1 illustrates the flow diagram of the study’s retrieval.

### 2.3. Data Collection Process

Data extraction was conducted using a customized table in Microsoft Excel. Table contents were authors and year, plant name, extract type, concentration of extract, method of extraction, vehicle type, cell type, stimulation time and order, assay, control, outcome, and viability assessment. Moreover, outcomes of each study were extracted according to the following categories: bioassay, outcome, highest non-cytotoxic, and lowest effective concentrations.

### 2.4. Quality Assessment

Assessment of evidence quality was conducted using the Toxicological data reliability assessment tool (ToxRtool, version 2009), which consists of fifteen items that assess various aspects of experimental studies [22]. These include chemical characterization of the material used, medium/vehicle, system/cell type, justification of the test system, test system preparation and maintenance, concentration, exposure duration, replication, the use of controls, endpoint, validity of method, data documentation and analysis, results reproducibility, study design clarity, and the plausibility of study design and data. Judgment for each item of the tool was scored as 0 if the study failed to meet the criterion, 1 if the study met the criterion, and N/A if the criterion did not apply to the study. According to the assessment tool, a score equal to or above 12 was considered reliable without restrictions, a score between 8 and 11 was considered reliable with restrictions, and a score below 8 was considered unreliable. Two examiners (IR and RG) conducted the quality assessment independently, and discrepancies were discussed until a consensus was reached.

### 2.5. Synthesis Method

Studies that met the eligibility criteria and were without full-text restrictions were included for synthesis. Data were extracted and tabulated for narrative synthesis. Comparison of outcomes was narratively reported to highlight potential variables that influenced the anti-inflammatory effects of the plant extracts, such as the type of vehicle and the extraction method. Furthermore, data from the inflammatory model and cell types were used to qualitatively contrast the single-cell inflammatory model against co-culture models.

## 3. Results

### 3.1. Study Characteristics and Extract Preparation

Twelve in vitro studies published between 2013 and 2022 met the inclusion criteria. Crude plant extracts were prepared from 13 species, including *Camellia sinensis*, *Salvia officinalis*, *Paeonia suffruticosa*, *Houttuynia cordata*, *Listea japonica*, *Populus nigra*, *Populus canadensis*, *Populus balsamifera*, *Theobroma cacao*, *Equisetum arvense*, *Alpinia katsumadai*, *Juncus effusus*, and *Zingiber officinale*. Extraction methods were primarily aqueous (*n* = 7) or ethanolic (*n* = 4), with one study using methanol. Extraction temperatures varied from room temperature (RT) to 95 °C, with specific temperatures reported for *Salvia officinalis* (95 °C), *Populus* spp. (60 °C), and *Juncus effusus* (60 °C). Purification techniques included microfiltration (0.2–0.45 µm) and centrifugation (1200–3000× *g*, 10–15 min) to ensure sterility and remove particulate matter. Commercial formulations (e.g., *Equisetum arvense*) employed H_2_O with butylene glycol as the vehicle. Stock concentrations (SC) ranged from 1% to 200%, while working concentrations (WC) were selected to maximize anti-inflammatory effects without compromising cell viability, typically in the micromolar or sub-percentage range (0.00004–10%). Several studies used multiple working concentrations to establish dose–response relationships. Table 1 summarizes the characteristics of included studies.

### 3.2. Cell Models and Inflammatory Stimulation

The studies employed a variety of oral cell models, including oral epithelial cell lines, primary gingival fibroblasts, primary periodontal ligament fibroblasts, keratinocytes, and monocytic cell lines (THP-1, RAW264.7). Inflammatory stimuli were mainly bacterial LPS from *P. gingivalis*, *A. actinomycetemcomitans*, *F. nucleatum*, or multiple oral pathogens. Other stimuli included phorbol 12-myristate 13-acetate (PMA) and silver nanoparticles (AgNPs). Several studies assessed basal inflammation without exogenous stimuli. Treatment protocols varied: extracts were applied prior to, simultaneously with, or following inflammatory stimulation, with durations ranging from 30 min to 24 h.

### 3.3. Cell Viability and Cytotoxicity

Most studies incorporated cytotoxicity assessments to confirm extract safety at tested concentrations. Assays included MTT (*n* = 7), WST-1 (*n* = 1), MTS (*n* = 1), and CellTiter-Blue (*n* = 1). Generally, extracts were non-cytotoxic across working concentrations, with viability maintained above 80–90% in most models. Notably, Satthakarn et al. (2015) [27] and Shiba et al. (2021) [31] did not perform viability assays, and one study used relatively high working concentrations that may have affected cell health. These data confirm that effective anti-inflammatory concentrations were largely safe for oral cells in vitro.

### 3.4. Anti-Inflammatory Effects

Crude plant extracts consistently demonstrated anti-inflammatory activity across all studies. *Camellia sinensis* (aqueous) reduced IL-6, IL-8, and CCL5 in oral epithelial cells, while *Salvia officinalis* (aqueous, 95 °C) suppressed IL-6 and IL-8 in gingival fibroblasts. *Paeonia suffruticosa* (ethanolic) downregulated CXCL9, CXCL10, and CXCL11 in fibroblasts, and *Houttuynia cordata* (aqueous) attenuated basal CXCL5 and CCL2 in primary gingival epithelial cells. *Listea japonica* (ethanolic) decreased IL-6 and IL-8 in periodontal ligament fibroblasts exposed to multiple LPS species, effective in both pre-treatment and co-treatment protocols. *Populus* spp. (methanolic) reduced IL-6 and IL-1β in fibroblasts challenged with silver nanoparticles. *Theobroma cacao* (ethanolic) lowered IL-6 and IL-8 in epithelial cells stimulated with *F. nucleatum*, while *Equisetum arvense* (commercial H_2_O + butylene glycol formulation) suppressed TNF-α in keratinocyte and monocytic cells. *Alpinia katsumadai* (ethanolic) attenuated COX-2 and PGE_2_ in fibroblasts, keratinocytes, and macrophages under LPS stimulation; *Juncus effusus* (aqueous, 60 °C) decreased IL-8 and CCL20 in keratinocytes, and *Zingiber officinale* (aqueous, RT) reduced IL-6 and IL-8 in primary gingival fibroblasts when applied both before and after LPS exposure. These findings indicate that crude plant extracts, irrespective of extraction method, effectively modulate key pro-inflammatory cytokines and chemokines across diverse oral cell types at cytocompatible concentrations. Table 2 summarizes the outcomes of the included studies.

### 3.5. Quality Assessment Results

Quality assessment of the collected studies highlights the reliability of the presented evidence. The mean ToxRTool score across studies of this review was 12.6 ± 1.7 (range 9–15), indicating moderate to high reliability. Eight studies (66.6%) were classified as reliable without restrictions, while four studies (33.3%) were reliable with restrictions. Six criteria were fulfilled by all studies: test substance administration, test system (cell culture identification), test system preparation and maintenance, exposure duration, endpoints measured, and data documentation and statistical analysis. Extract identification and replicates had the lowest fulfillment rates (33.3% and 58.3%, respectively). While these findings show a significant reliability of the evidence supporting the anti-inflammatory potential of crude plant extracts, they underscore persistent methodological shortcomings in reporting plant extract behavior within in vitro oral inflammation studies.

**Figure 2:**
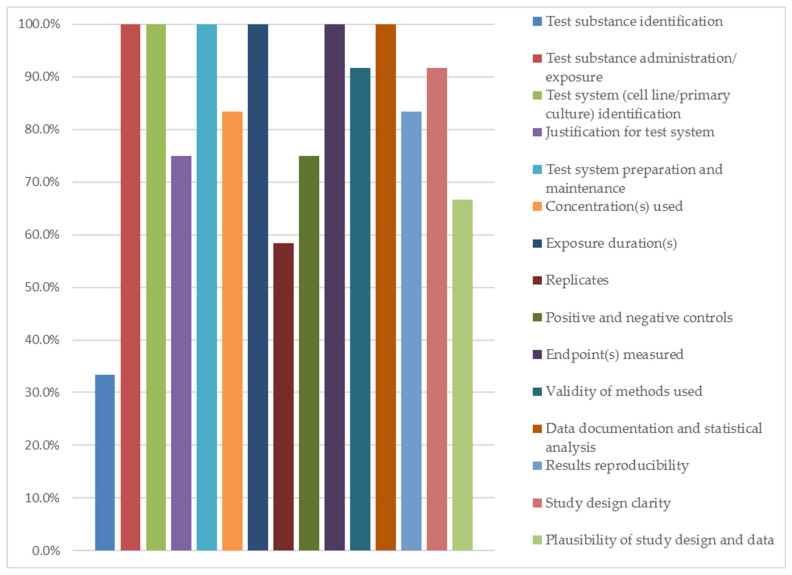
Studies’ compliance rate with the ToxRTool criteria.

Table 3 presents scores and descriptive statistics of quality assessment for studies in this review. Figure 2 illustrates the compliance rate of studies with the ToxRTool assessment criteria. Full details of quality assessment can be found in Table S1 in the Supplementary Materials.

## 4. Discussion

To date, no systematic reviews have specifically examined the anti-inflammatory potential of crude plant extracts in oral inflammatory cell models. By consolidating in vitro evidence, this review demonstrates that unrefined plant preparations exhibit measurable anti-inflammatory activity across different oral cell types while maintaining cytocompatibility. These findings support the pharmacological validity of traditional herbal practices and identify key methodological challenges that must be addressed for clinical translation.

While this review underscores the limited evidence supporting the anti-inflammatory role of plant extract in oral inflammation, it also reveals important methodological shortcomings of the existing literature. Among the included studies, four—those by Yun et al. (2013) [25], Satthakarn et al. [27], Shin et al. [32], and Al-Shibani et al. [34]—were deemed reliable with restrictions. Despite most studies demonstrating general reliability, only four—Ehrnhöfer-Ressler et al. [24], Yun et al. (2013) [25], Pobłocka-Olech et al. [29], and Ben Lagha et al. [30]—reported adequate characterization of their plant extracts, making this the most frequently unmet criterion in the ToxRTool assessment. While in vitro investigations of crude plant extracts provide valuable insights into their cytoprotective, antioxidant, and anti-inflammatory properties, chemical characterization is crucial for identifying the active constituents responsible for these effects. As highlighted by Brusotti et al., characterization methods should be tailored to the intended biological activity to enhance pharmacological relevance and reduce unwanted metabolites [35].

The herbal extracts investigated in this review consisted of plant species from ten botanical families. Table 4 summarizes the characteristics of plant extracts investigated across studies of this review. The diverse taxonomical and phytochemical properties of the plants underscore their diverse biological activities. The broad pharmacological spectrum, on the other hand, indicates that the putative anti-inflammatory effects of the reported plant extracts are possibly a result of integrative cellular mechanisms that eventually mask inflammation. Chemical characterization and mechanistic investigation of inflammatory signaling promote a targeted therapeutic potential. For instance, polyphenol-driven IL-1β and TNF-α inhibition from *Camellia sinensis* and *Salvia officinalis* offers protective antioxidant effects to lessen the inflammatory sequelae of oxidative stress. Whereas salicylates-mediated reduction of COX1 and prostaglandin from *Populus* spp. offers analgesic effects.

Another recurrent methodological weakness observed in the included studies was the inconsistent use of experimental replicates. Specifically, five studies—those by Yun et al. (2013) [25], Lombardo Bedran et al. [26], Yun et al. (2018) [28], Shin et al. [32], and Al-Shibani et al. [34]—lacked replicates in their inflammatory bioassays. The absence of replication limits data reliability and increases the risk of misinterpreting cytostatic or anti-proliferative effects as genuine anti-inflammatory responses. In addition, many studies reported the anti-inflammatory effects of plant extracts without a dose–response relationship, which is crucial in the validation of the evidence and balancing the efficacy with cytocompatibility.

Across the included studies, aqueous and ethanolic extracts consistently reduced the expression and secretion of pro-inflammatory mediators such as IL-1β, IL-6, and IL-8, as well as chemokines including CCL2, CCL5, and CXCL family members. These effects occurred at non-cytotoxic concentrations—typically between 10 and 125 µg/mL—indicating genuine biological activity rather than metabolic suppression. Collectively, these observations affirm that crude plant extracts can modulate inflammatory signaling in oral cells, supporting their non-cytotoxic anti-inflammatory potential and possibly their efficacy as adjuncts for managing oral inflammation.

The oral cavity represents a complex microenvironment in which epithelial cells, fibroblasts, keratinocytes, immune cells, and the oral microbiome interact to maintain tissue integrity and immune balance [48]. In periodontitis, this balance is disrupted, leading to an excessive cytokine-driven response and tissue destruction [49,50]. Similarly, in oral mucositis, exposure of the oral mucosal tissues to radiation results in damage signals from keratinocytes, tissue fibroblasts, and endothelial cells, resulting in painful inflammation and ulceration of the mucosal tissue [51,52]. Additionally, both in periodontitis and oral mucositis, resident immune cells participate in perpetuating the inflammatory response [53,54,55,56]. Collectively, these observations demonstrate how the interplay of oral cells maintains homeostasis and supports their employment in in vitro studies of oral inflammatory conditions.

The consistency of the test systems employed across studies in this review reinforces the evidence for the anti-inflammatory potential of crude plant extracts in oral inflammation. In vitro models using oral fibroblasts and epithelial cells have proven valuable for mechanistic investigations; however, they often fail to reproduce the cellular interplay characteristic of in vivo inflammation. Kasurinen et al. and Karri et al. reported that co-culture models elicited stronger inflammatory responses compared to monocultures [57,58]. Therefore, future research should prioritize co-culture or three-dimensional models incorporating immune cells to more accurately simulate the inflammatory cascade, enabling more physiologically relevant testing of plant-derived therapeutics. Notably, none of the studies included in this review employed a co-culture model.

On the other hand, oral keratinocytes are a fundamental cell type within the oral mucosa, contributing to both inflammatory signaling and epithelial repair. Pleguezuelos et al. demonstrated that gingival keratinocytes act as early responders to microbial LPS, mediating inflammation through MAP-dependent IL-1ß release [59]. In contrast, Hujiahemaiti et al. reported that a plant-derived compound enhanced oral keratinocyte proliferation, underscoring their importance in tissue regeneration and wound healing [60]. These findings illustrate the methodological variability in how keratinocytes are employed across in vitro models of oral inflammation.

In the context of gingivitis and periodontitis, keratinocytes play a limited role due to their relative scarcity within the junctional epithelium. Conversely, they are functionally prominent in regenerative processes, particularly in the masticatory mucosa, where keratinization significantly influences healing dynamics [61,62]. Therefore, their employment within in vitro studies of oral mucositis is physiologically justified. Only two studies within this review, those by Wada et al. and Shiba et al., incorporated keratinocytes, highlighting a potential methodological gap and the need for purposeful employment of cell types for the studied model of inflammation.

Despite differences in cell type and inflammatory stimuli, methodological consistency across studies was relatively high. Most investigations used bacterial LPS or oral pathogens to elicit inflammation. Studies by Ehrnhöfer-Ressler et al. and Pobłocka-Olech et al. have utilized PMA and AgNP as inflammatory stimuli, respectively, while Satthakarn et al. did not use a positive control [24,27,29]. While chemical stressors induce cellular damage signals, it has been demonstrated that their elicited effects differ from those produced by cytokines and LPS driven by exposure to oral pathogens [63,64]. On the other hand, methods of quantification of cytokine modulation were consistent across studies in this review. They consisted primarily of ELISA or RT-PCR. These assays remain the benchmark for assessing inflammatory outcomes, while multiplex technologies offer higher throughput, provided they are validated against ELISA [65,66,67,68]. Two studies in this review, by Ehrnhöfer-Ressler et al. and Al-Shibani et al., utilized the multiplex immunoassay in their experiments [24,34]. The adoption of standardized stimulation protocols, treatment sequences, and endpoint markers would substantially enhance reproducibility and permit quantitative comparisons across studies.

Variability in extraction methods represents a key determinant of outcome differences [69,70]. In this review, aqueous extractions were predominant, followed by ethanolic or methanolic techniques. Because solvent polarity directly affects the yield and spectrum of bioactive compounds, solvent selection must align with the physicochemical properties of target phytochemicals [71]. Organic solvents often enhance recovery of phenolics and flavonoids, while water-based extractions remain more clinically relevant for topical and oral applications [72]. Systematic reporting and standardization of extraction parameters are therefore essential to ensure reproducibility and facilitate clinical translation. Nonetheless, Jeyaraj et al. reported that aqueous extraction results in a bioactive yield regardless of the solvent type [73]. Therefore, in the context of practical infusion preparations, the use of water as a solvent may also be effective in obtaining the therapeutic benefits of the plant material.

For centuries, herbal materials have been used in traditional medicine to treat a plethora of human ailments, and they continue to serve as accessible therapeutic options in regions with limited healthcare resources [74,75]. Plant-based medicines offer a safer alternative to synthetic anti-inflammatory drugs, demonstrating comparable efficacy to NSAIDs but with fewer adverse effects [76,77,78]. While the effects of plant-based therapies are not as rapid and potent as synthetic drugs, their efficacy with fewer side effects outweighs this limitation. In the context of oral inflammation, their topical application provides a practical advantage, enabling local relief without systemic absorption. Conventional topical therapies, though effective, may cause adverse outcomes such as fungal infections or systemic side effects with prolonged use [79]. Conversely, plant-derived formulations—such as curcumin, honey, and chamomile—have shown promise in reducing the severity of oral inflammatory conditions such as radiation mucositis [80,81]. These formulations exhibit favorable safety profiles compared to synthetic products such as chlorhexidine [82,83]. Although current clinical evidence remains mixed due to variability in herbal formulations and study design, larger standardized trials are warranted to confirm their therapeutic efficacy and safety in oral inflammation.

Safety, however, remains a prerequisite for translation. While most studies reported high cytocompatibility, metabolic assays such as MTT alone do not fully capture sub-lethal or long-term effects [84]. Cellular effects of plant extracts transcend metabolic alteration to potentially impose alterations in junctional adherence, membrane integrity, gene toxicity, and, in a simpler context, assay interferences [84,85,86]. Therefore, comprehensive toxicity testing under GLP conditions, including membrane integrity and genotoxicity assessments, is necessary to establish safety profiles [87]. Additionally, natural variability arising from plant source, harvest time, and extraction technique must be controlled through standardized sourcing and analytical characterization of bioactive constituents [88]. Jantan et al. suggested that the clinical translation of effective immunomodulatory plant-derived compounds requires improved quality and standardization of extraction methods to improve their bioavailability and minimize cytotoxicity [89].

Early mechanistic evidence suggests that many crude extracts modulate inflammation through inhibition of the NF-κB signaling pathway, a central regulator of inflammatory gene expression [90,91,92]. In this review, *Theobroma cacao* and *Equisetum arvense*, investigated by Ben Lagha et al. and Shiba et al., respectively, demonstrated NF-κB–dependent anti-inflammatory effects in oral cells, aligning with broader findings across other plant-derived therapeutics [30,31]. Research interest in exploring this role resurged in the late 2010s, when studies began to employ sophisticated molecular approaches, such as gene expression and pathway-level analysis, to elucidate how plant extracts exert their anti-inflammatory effects via the NF-κB signaling system [93,94]. Future investigations integrating transcriptomic or proteomic approaches could unravel synergistic mechanisms among phytochemicals and clarify their collective impact on inflammatory networks.

Studies within this review demonstrated a notable degree of homogeneity in both endpoints and measurement methods. The consistent quantification of key pro-inflammatory cytokines (IL-1ß and IL-6) and chemokines (CCL5 and IL-8) using ELISA strengthens the reliability of the findings by aligning with standardized inflammatory bioassays [65]. However, the selective measurement of only a few cytokines and chemokines may limit the mechanistic depth of interpretation. Notably, IL-8 is a pleiotropic chemokine that can be upregulated by diverse cellular events such as proliferation, angiogenesis, chemical injury, and oxidative stress [95,96]. Thus, relying solely on quantitative cytokine assessment represents a methodological limitation. Integrating signaling pathway analyses—such as NF-κß, MAPK, or Nrf2 activation—would provide deeper mechanistic insight into the molecular basis of the anti-inflammatory effects exerted by plant extracts.

An exception in the endpoints measured was the study by Shin et al., which incorporated prostaglandin E2 (PG-E2) and COX2 as outcome markers for the anti-inflammatory effects of *Alpinia katsumadai*. Cytokines and chemokines such as IL-6 and IL-8 are considered more robust markers of inflammatory activation due to their rapid induction and greater fold changes compared to PGE2 and COX2 [97,98]. This is due to PGE2 and COX2 being more constitutive in some tissues, reflecting their physiological role in tissue homeostasis.

While this systematic review provides supportive evidence for the anti-inflammatory potential of plant extracts in oral inflammatory cell models, its conclusions should be interpreted carefully due to several caveats. First, the review exclusively includes in vitro studies investigating crude plant extracts rather than isolated bio-compounds, which may miss the mechanistic clarity and specificity offered by isolated compounds [99]. In addition, collecting studies exclusively from the PubMed database may narrow the evidence of the anti-inflammatory potential. Therefore, comprehensive future work is needed to amalgamate the evidence and to explore in depth the methodological limitations of crude plant extracts in in vitro studies. Using crude plant extracts reflects a broader therapeutic application and aligns with traditional medicinal and ethnobotanical practice; however, the observed effects of crude plant extracts may vary across different cell models due to potential interferences between plant components and the biological assay system [100]. Moreover, the reproducibility of in vitro studies that utilize crude plant extracts can be lower than those employing isolated compounds, owing to variability in plant source, growth conditions, and extraction methods [101]. Finally, the heterogeneity in extraction methods, stimulation conditions, and cell systems prevents the conduction of a meta-analysis, which limits evidence generalizability.

## 5. Conclusions

In conclusion, crude plant extracts emerge as promising, cytocompatible, and biologically active agents capable of modulating key inflammatory mediators in oral tissues. Future research that employs complex models, such as co-culture or 3D models, and standardized experimental design using well-characterized plant extracts is needed. Bridging ethnobotanical knowledge with standardized experimental design, mechanistic analysis, and rigorous safety validation is crucial for translating these natural materials into evidence-based therapeutics. Such an approach may ultimately yield accessible, sustainable, and safe alternatives to conventional anti-inflammatory drugs for the management of oral inflammatory diseases.

## Figures and Tables

**Table 1:** Characteristics of included studies.

Author and Year	Plant Name	Extract Type	Extraction Temperature	Concentration	Medium/Vehicle	Purity	Cell Type	Inflammatory Stimulus	Stimulation Time/Order	Cell Viability Assessment
Zhao et al., 2013 [23]	*Camellia sinensis*	aqueous	RT	SC: 2% WC:0.01%, 0.005%, 0.0025%, 0.0001%	D.H_2_O	0.45 µm filter	Oral epithelial cell line	*P. gingivalis* extract	Extract for 2 h, then 24 h after stimulation	MTT
Ehrnhöfer-Ressler et al., 2013 [24]	*Salvia officinalis*	aqueous	95 °C	SC: 1% WC: 1%	H_2_O	none	Gingival fibroblast cell line	PMA	Stimulation for 6 h, then plant extract for 6 h	WST-1
Yun et al., 2013 [25]	*Paeonia suffruticosa*	ethanolic	RT	SC: 10% WC: 4%	DMSO	none	Primary gingival fibroblasts	LPS	Extract for 1 h, then LPS for 24 h	MTS
Lombardo Bedran et al., 2015 [26]	*Camellia sinensis*	aqueous	-	SC: 4% WC: 0.02% 0.01%	D.H_2_O	0.2 µm filter	Oral epithelial cell line	LPS (*A. actinomycetemcomitans)*	Extract for 2 h, then stimulation for 24 h	MTT
Satthakarn et al., 2015 [27]	*Houttuynia cordata*	aqueous	-	SC: not mentioned WC: 0.02%, 0.01%, 0.005%, 0.0025%	H_2_O	none	Primary gingival epithelial cells	none	Extract for 18 h	CellTiter-Blue
Yun et al., 2018 [28]	*Listea japonica*	ethanolic	RT	WC: 0.01%	ethanol	none	Primary PDL fibroblast	LPS (*P. gingivalis*, *T. forythea*, *T. denticola*, *F. nucleatum)*	Dual (extract + LPS) for 0, 1, 3, 6, 12, 24 h Pre-treatment with the extract for 2 h, then LPS for 24 h	MTT
Pobłocka-Olech et al., 2019 [29]	Populus spp.: *P. nigra*, *P. berolinensis*, *P. lasiocarpa*	methanolic	60 °C	SC: 5% WC: P.n + P.l: 0.0015% P.b: 0.00075%	methanol	none	Gingival fibroblast cell line	AgNP	Extract for 1 h, then stimulation for 18 h	MTT
Ben Lagha et al., 2021 [30]	*Theobroma cacao*	ethanolic	-	SC: 2% WC: 0.025% 0.0125% 0.0063%	DMSO	0.22 µm filter	Oral epithelial cell lines	*F. nucleatum*	Extract for 30 min, then stimulation for 24 h	MTT
Shiba et al., 2021 [31]	*Equisetum arvense*	-	-	WC: 0.00004%	Commercially formulated: H_2_O + butylene glycol	none	Oral keratinocyte cell line, monocytic cell line	LPS (*P. gingivalis*, *A. actinomycetemcomitans)*	Co-stimulation for 6 h	None
Shin et al., 2021 [32]	*Alpinia katsumadai*	ethanolic	-	WC: 0.001%	ethanol	none	Gingival fibroblast and oral keratinocyte cell lines, murine macrophage cell line	LPS (dental plaque, *P. gingivalis*)	Co-stimulation for 24 h	MTT
Wada et al., 2022 [33]	*Juncus effusus*	aqueous	60 °C	SC: 200% WC: 10% 2%	Ultra-pure H_2_O	0.45 µm filter + centrigation 1200× *g* 15 min	Oral keratinocyte cell line	LPS (*P. gingivalis)*	Co-stimulation for 24 h, extract for 4 h, then stimulation for 24 h	None
Al-Shibani et al., 2022 [34]	*Zingiber officinale*	aqueous	RT	SC: 20% WC: 0.005%	D.H_2_O	0.22 µm filter + centrifugation 3000× *g* 10 min	Primary gingival fibroblast	LPS (*P. gingivalis*)	Extract for 24 h, then LPS for 24 h, and vice versa	MTT

Abbreviations: RT—room temperature; SC—stock concentration; WC—working concentration; PMA—phorbol 12-myristate 13-acetate; LPS—lipopolysaccharide; MTT—3-[4;5-dimethylthiazol-2-yl]-2;5 diphenyl tetrazolium bromide; WST-1—water-soluble tetrazolium salts; and MTS—(3-(4;5-dimethylthiazol-2-yl)-5-(3-carboxymethoxyphenyl)-2-(4-sulfophenyl)-2H-tetrazolium).

**Table 2:** Summary of bioassay outcomes.

Author and Year	Assay	Outcome	Extract Highest and Non-Cytotoxic Concentration	Extract Lowest Effective Concentration
Zhao et al., 2013 [23]	ELISA	↓ CCL5, IL-6, IL-8	100 µg/mL	25 µg/mL
Ehrnhöfer-Ressler et al., 2013 [24]	Multiplex immunoassay	↓ IL-6, IL-8	NDR	NDR
Yun et al., 2013 [25]	RT-PCR	↓ CXCL9, CXCL10, CXCL11	NDR	NDR
Lombardo Bedran et al., 2015 [26]	ELISA	↓ IL-8	200 µg/mL	100 µg/mL
Satthakarn et al., 2015 [27]	RT-PCR ELISA	↓ CXCL5 ↓ CCL2	200 µg/mL	25 µg/mL
Yun et al., 2018 [28]	RT-PCR ELISA	↓ IL-6, IL-8 ↓ IL-6, IL-8	100 µg/mL	10 µg/mL
Pobłocka-Olech et al., 2019 [29]	RT-PCR ELISA	↓ IL-6, IL-1ß ↓ IL-6, IL-1ß	NDR	NDR
Ben Lagha et al., 2021 [30]	ELISA	↓ IL-6, IL-8	250 µg/mL	125 µg/mL
Shiba et al., 2021 [31]	RT-PCR ELISA	↓ TNF-α ↓ secretion of TNF-α	NDR	NDR
Shin et al., 2021 [32]	ELISA	↓ PG-E2, COX-2	NDR	NDR
Wada et al., 2022 [33]	ELISA	↓ IL-8, CCL-20	NDR	NDR
Al-Shibani et al., 2022 [34]	xMap Milliplex	↓ IL-1ß, IL-8	NDR	NDR

Abbreviations: ↓ decreased, NDR—no dose–response.

**Table 3:** Summary and statistics of quality assessment of studies.

Author	Score	Comment
Zhao et al., 2013 [23]	**14**	reliable without restrictions
Ehrnhöfer-Ressler et al., 2013 [24]	**14**	reliable without restrictions
Yun et al., 2013 [25]	**11**	reliable with restrictions
Lombardo Bedran et al., 2015 [26]	**13**	reliable without restrictions
Satthakarn et al., 2015 [27]	**9**	reliable with restrictions
Yun et al., 2018 [28]	**13**	reliable without restrictions
Pobłocka-Olech et al., 2019 [29]	**14**	reliable without restrictions
Ben Lagha et al., 2021 [30]	**15**	reliable without restrictions
Shiba et al., 2021 [31]	**13**	reliable without restrictions
Shin et al., 2021 [32]	**11**	reliable with restrictions
Wada et al., 2022 [33]	**13**	reliable without restrictions
Al-Shibani et al., 2022 [34]	**11**	reliable with restrictions
Mean	**12.6**	
Median	**13**	
Standard Deviation	**1.7**	
Range	**6**	
Minimum	**9**	
Maximum	**15**	
Confidence Level (95.0%)	**1.099**	

**Table 4:** Summary of characteristics of plant extracts.

Plant Species (Common Name)	Family	Major Phytochemical Groups	Principal Anti-Inflammatory Mechanisms
*Camellia sinensis* (Green tea) [36]	Theaceae	Polyphenols (catechins, EGCG, theaflavins)	Antioxidant; inhibits NF-κB, COX-2, and iNOS; activates Nrf2
*Salvia officinalis* (Sage) [37]	Lamiaceae	Rosmarinic acid, carnosic acid, flavonoids, terpenes	NF-κB and MAPK suppression; COX-2 inhibition; antioxidant
*Paeonia suffruticosa* (Tree peony) [38]	Paeoniaceae	Paeonol, paeoniflorin, flavonoids	Inhibits MAPK and NF-κB; reduces IL-6, TNF-α; antioxidant
*Houttuynia cordata* (Chameleon plant) [39]	Saururaceae	Flavonoids, polysaccharides, and volatile oils	Immunomodulatory; suppresses IL-6, TNF-α, and NO; antiviral
*Litsea japonica* [40,41]	Lauraceae	Monoterpenes, flavonoids, lignans	Inhibits NO and TNF-α production; antioxidant
*Populus* spp. (poplar) [42]	Salicaceae	Phenolic glycosides (salicin, populin), flavonoids	COX inhibition; prostaglandin synthesis suppression
*Theobroma cacao* (Cocoa) [43]	Malvaceae	Polyphenols (procyanidins), theobromine, flavanols	Antioxidant; inhibits TNF-α, IL-6; enhances NO for vascular repair
*Equisetum arvense* (Horsetail) [44]	Equisetaceae	Silica, flavonoids, phenolic acids	Antioxidant; mild cytokine suppression; enhances collagen synthesis
*Alpinia katsumadai* (*Katsumada galangal*) [45]	Zingiberaceae	Diarylheptanoids, flavonoids, terpenes	Inhibits NO, COX-2, and IL-6; modulates MAPK
*Juncus effusus* (Soft rush) [46]	Juncaceae	Phenanthrenes, flavonoids, polysaccharides	Suppresses TNF-α, IL-6; antioxidant
*Zingiber officinale* (Ginger) [47]	Zingiberaceae	Gingerols, shogaols, zingerone	COX and LOX inhibition; NF-κB suppression; antioxidant

Abbreviations: EGCG—epigallocatechin gallate; iNOS—inducible nitric oxide synthase; NO—nitric oxide.

## Data Availability

The raw data supporting the conclusions of this article will be made available by the authors on request.

## Supplementary Materials

The following supporting information can be downloaded at: https://www.mdpi.com/article/10.3390/ijms262311253/s1.

## Abbreviations

The following abbreviations are used in this manuscript:

MeSH	medical subject headings
NDR	no dose–response
PG-E2	prostaglandin-E2
COX2	cyclooxygenase-2
RAS	recurrent apthous stomatitis
PRISMA	preferred reporting items for systematic reviews and meta-analysis
PMA	phorbol 12-myristate 13-acetate
AgNP	silver nanoparticles
MTT	3-[4,5-dimethylthiazol-2-yl]-2,5 diphenyl tetrazolium bromide
RT	room temperature
SC	stock concentration
WC	working concentration
D.H_2_O	distilled water
DMSO	dimethyl sulfoxide
LPS	lipopolysaccharide
ELISA	enzyme-linked immunosorbent assay
RT-PCR	real-time polymerase chain reaction
WST-1	water-soluble tetrazolium salts
MTS	(3-(4,5-dimethylthiazol-2-yl)-5-(3-carboxymethoxyphenyl)-2-(4-sulfophenyl)-2H-tetrazolium)
